# Genome-Wide Characterization and Functional Analysis of ABCG Subfamily Reveal Its Role in Cutin Formation in Cotton

**DOI:** 10.3390/ijms24032379

**Published:** 2023-01-25

**Authors:** Xuehan Huo, Ao Pan, Mingyang Lei, Zhangqiang Song, Yu Chen, Xin Wang, Yang Gao, Jingxia Zhang, Shengli Wang, Yanxiu Zhao, Furong Wang, Jun Zhang

**Affiliations:** 1 Life Science College, Shandong Normal University, Jinan 250358, China; 2 Key Laboratory of Cotton Breeding and Cultivation in Huang-Huai-Hai Plain, Ministry of Agriculture and Rural Affairs, Institute of Industrial Crops, Shandong Academy of Agricultural Sciences, Jinan 250100, China; 3 College of Bioscience & Biotechnology, Hunan Agricultural University, Changsha 410128, China

**Keywords:** *Gossypium*, ABCG, function, cutin formation, ultrastructure, VIGS

## Abstract

ATP-binding cassette transporter G (ABCG) has been shown to be engaged in export of broad-spectrum compounds with structural differences, but little is known concerning its role in cutin formation of cotton (*Gossypium* spp.). In this study, we conduct a genome-wide survey and detected 69, 71, 124 and 131 ABCG genes within *G. arboretum*, *G. raimondii*, *G. hirsutum* and *G. barbadense*, separately. The above ABCGs could be divided into four groups (Ia, Ib, Ic, II). Some *ABCG* genes such as *GhABCG15*, whose homologous gene transports cuticular lipid in *Arabidopsis*, was preferentially expressed in the development of fiber. A weighted gene co-expression network analysis (WGCNA) demonstrated that *GhABCG* expression was significantly associated with the amount of 16-Hydroxypalmitate (a main component of cutin precursor) in cotton fibers. Further, silencing of *GhABCG15* by virus-induced gene silencing (VIGS) in cotton generated brightened and crinkled leaves as well as reduced thickness of cuticle and increased permeability. Chemical composition analysis showed the cutin content in *GhABCG15*-silenced leaves had decreased while the wax content had increased. Our results provide an insight for better understanding of the role of the *Gossypium* ABCG family and revealed the essential role of GhABCGs in cotton cutin formation.

## 1. Introduction

ATP-binding cassette (ABC) transporters can be detected in organisms of diverse kingdoms and exert a variety of functional roles through the transport of various chemicals in and out of bio-membranes as well as through exerting transport-unrelated biochemical activities [1,2,3]. The full-size type includes many ABC transporter proteins encoding fully functional units, containing two transmembrane domains (TMD) that can generate a core region and two nucleotide binding domains (NBD) that can generate an outer cytosolic region [4]. However, the half-sized transporters included one TMD and one NBD [5]. Of all ABC transporters, only the ABCG subfamily possesses a reverse organization of domains on the polypeptide chains of full (PDR proteins or Pleotropic Drug Resistance) as well as half-sized (White-Brown complex or WBC proteins) proteins [5,6]. Compared to other subfamilies of ABC proteins, the G subfamily members are associated with more duplication and proliferation in plants [7,8]. It has been reported that ABCGs are involved in several functions, such as transport of abscisic acid (ABA) [9], sporopollenin [10], lignin precursors [11] and cuticular lipids [12,13].

As a protective hydrophobic layer including outer aerial plant tissue surface, the plant cuticle exerts vital functions such as maintaining water balance between the plant and the environment [14,15], defensing against UV radiation and enhancing resistance to pests or pathogens [16,17], as well as providing a highly hydrophobic barrier that suppresses plant-adjacent organ fusion [18]. To be specific, the plant cuticle is a complex architecture including the cuticle proper, intra-cuticular wax and epicuticular wax [19]. Cutin primarily includes oxygenated C16/C18 fatty acids (FAs), with low non-oxidized FA contents, fatty aldehydes and dicarboxylic acids, with lesser amounts of glycerol and phenylpropanoids [20], while wax contains very long chain fatty acids (VLCFAS) (dominant chain length, 20-34 carbons) and their derivatives containing fatty ketones, fatty alcohols, alkanes, aldehydes, triterpenoids, flavonoids and wax esters [15,21]. Wax and cutin precursors once synthesized eventually cross through the polysaccharide cell wall and plasma membrane to the emerging cuticular membrane [22].

ATP-binding cassette (ABC) transporters exert a vital function in wax and cutin precursor trafficking in and out of plasma membrane. ABCG12 and ABCG11 have been identified as protein channels related to cuticular components’ transport in *Arabidopsis thaliana* and their mutants exhibit an array of surface defects and unusual lipidic cytoplasmatic inclusions in epidermal cells [13,23,24,25]. Additionally, AtABCG13/WBC13 has been associated with cuticle lipid secretion and this protein primarily mediates the transport of cutin precursors in *Arabidopsis* flowers, as well as affects the characteristics of the epidermal cells in the petals [26,27]. Additionally, it has been identified that a full-scale transporter, AtABCG32, makes a contribution to the forming of epicuticular nano-ridges on the petals of flowers. The *atabcg32* mutant exhibits defects in the nano-ridge and increases permeability of the cuticle [28].

Cotton (*Gossypium* spp.) refers to a vital cash crop producing natural fiber. In previous years, several studies have been documented in identifying the ABC genes family in cotton [29,30]. Nonetheless, the role of ABCG in cotton cutin formation still remains unknown. With a better understanding of the role of the *Gossypium* ABCG subfamily, genome-wide identification and evolutionary analysis were performed for ABCG in *G. raimondii*, *G. arboreum*, *G. hirsutum* and *G. barbadense*. Meanwhile, conserved domain, gene structure, transcription level promoters and motifs of *GhABCG* genes in diverse tissues and their responses upon abiotic stresses were further explored. Especially, expression patterns of *GhABCG*s were explored in cotton developing fiber and by conducting WGCNA we analyze the correlation between the co-expressed gene modules and lipid metabolites related to the synthesis of cutin and waxy precursors. We also investigated the function of GhABCG15 in the transport of lipids by silencing its expression in cotton with the use of virus-induced gene silencing (VIGS). Our study will be conducive to further exploration of the functional roles of *Gossypium* ABCGs during cotton development.

## 2. Results

### 2.1. Identification and Classification of ABCGs in Cotton

Forty-three *Arabidopsis* ABCG protein sequences retrieved from the TAIR database were applied as query in order to perform a blast search against the cotton genome database. Furthermore, we employed the Hidden Markov Model (HMM), built with *Arabidopsis* ABCG proteins, as queries to search the ABCGs in the four *Gossypium* species by Hmmersearch with default parameters. The protein sequences of identified *Gossypium* ABCGs through the above two pathways were exposed to SMART and Pfam tools for evaluation of the existence of TMD–NBD (WBC) or TMD–NBD–TMD–NBD(PDR) structures. As a result, 69, 71, 124 and 131 ABCG members were identified in *G. arboretum* (A genome), *G. raimondii* (D genome), *G. hirsutum* (AD genome) and *G. barbadense* (AD genome), separately (Supplementary Table S1). The protein length of *Gossypium* ABCG range from 717 (*GH_D11G2292*) to 5166 (*GH_D02G2156*), molecular weight from 26.62 to 190.79 kD and pI from 5.82 to 9.58. All *Gossypium* ABCG proteins are located on plasma membrane (Supplementary Table S2).

**Figure 1:**
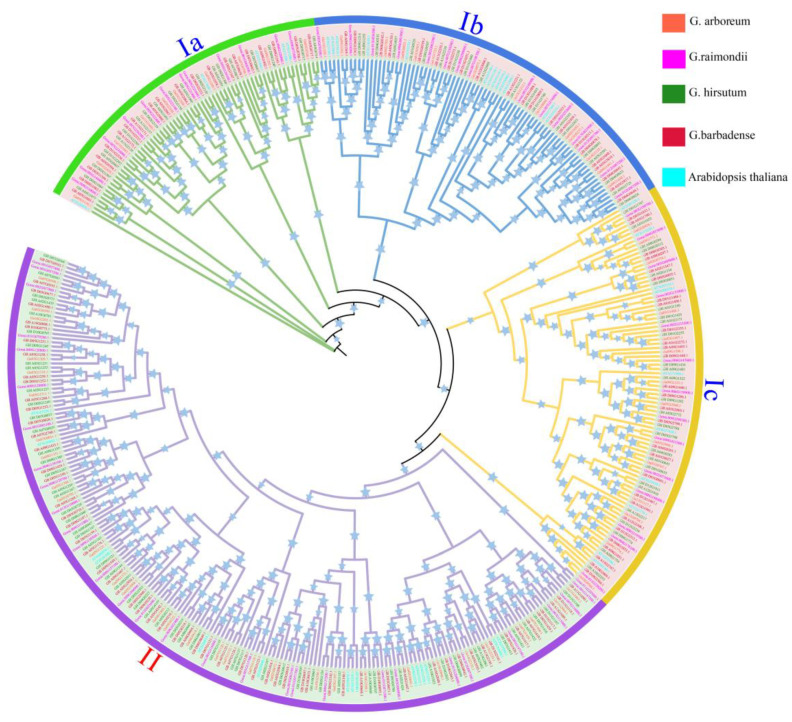
Phylogenetic tree of the ATP-binding cassette transporter G (ABCG) family in four Gossypium species and Arabidopsis. A total of 69, 71, 124 and 131 ABCG members identified in G. arboretum, *G. raimondii*, *G. hirsutum* and *G. barbadense*, respectively, together with 43 Arabidopsis ABCGs were submitted for phylogenetic analysis. Ia, Ib, Ic and II represent 4 subgroups.

To have an understanding of the evolutionary relationships of Gossypium ABCGs, a ML phylogenetic tree was constructed with the full length ABCG protein sequences from the four cotton species and Arabidopsis (Figure 1). Clearly, the Gossypium ABCGs were classified into four groups (Ia, Ib, Ic, II), in which Group Ia, Ib and Ic belong to WBC transporters and Group II belongs to PDR transporters. Group Ia, Ib and Ic contain 62, 70 and 83 members, respectively, and Group II includes 180 members. ABCGs from At and Dt of allotetraploids (G. hirsutum and G. barbadense) clustered with diploid G. arboretum and G. raimondii, separately, implying that the two subgroups evolved separately and were comparatively conserved in evolution.

### 2.2. Gene structure, Conserved Motif and Domain of GhABCGs

**Figure 2:**
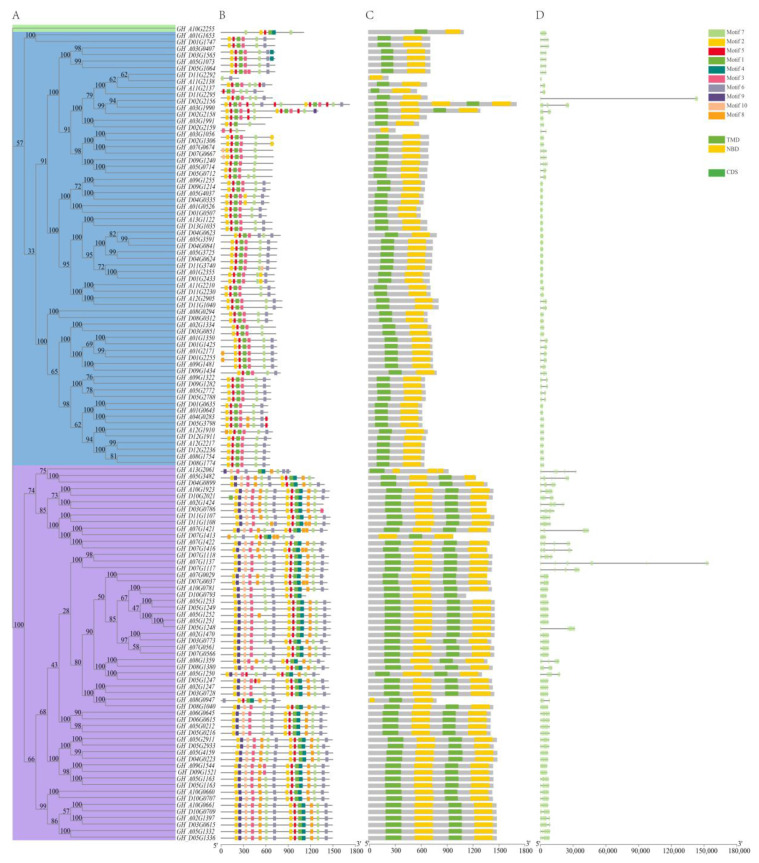
The schematic diagram of phylogenetic tree (**A**), conserved motif (**B**), domain (**C**) and exon/intron construction (**D**) of 124 ABCG members in *G. hirsutum*. (**A**) A total of 124 ABCGs in *G. hirsutum* were clustered into 3 clades shown in green, blue and purple, respectively. (**B**) and (**C**) The motifs or domains are indicated by colored boxes correspondingly. (**D**) The exon and intron of ABCGs are denoted by green box and gray line.

With the purpose of better understanding the similarity and diversity of the ABCG subfamily in *G. hirsutum*, gene structure and conserved motif of ABCG proteins were investigated (Figure 2). As expected, most *ABCG* genes with close evolutionary relationship exhibited very similar exon/intron distribution patterns in exon and intron number (Figure 2D) and the majority of *ABCG* members contain multiple exons and introns except for 16 *ABCG* genes, with no introns. Furthermore, 10 conserved motifs were detected with the use of the MEME program among the sequences of ABCG family nucleic acid (Figure 2B). The phylogenetic tree consists of three classes with 1, 66 and 57 genes, respectively (Figure 2A). The proteins of high homology have similar motifs. In the blue clade, all members contained motif 1, 2, 3, 5 and 7 except GH_D11G2292, GH_A11G2137 and GH_D02G2159, while in the purple clade, motif 1 to 10 are present in most ABCG proteins except GH_A13G2061, GH_D07G1413 and GH_A08G0947. GH_A10G2255 is clustered in a single clade. It is possible that there is motif loss during replication (Figure 2B). Consistent with the motif distribution, all ABCGs in the blue clade belonging to WBC contain a nucleotide-binding domain and a transmembrane domain except GH_D11G2292 and GH_D02G2159. Genes in the purple clade belonging to PDR include the double nucleotide-binding domain and the transmembrane domain except GH_A13G2061, GH_D07G1413 and GH_A08G0947 (Figure 2C).

### 2.3. Chromosomal Location, Duplication and Evolution

The physical localization of *ABCG* genes of four *Gossypium* species were analyzed by BLASTN searches against the chromosomal locations based on the cotton genome database. In addition, the obtained findings show that all of the *ABCG* genes are distributed unevenly across different chromosomes and, as expected, the homologous genes from At and Dt were symmetrically distributed in the sub-genome of two allotetraploid species (Supplementary Figure S1). Chromosomes At_05 and Dt_05 have a higher number of ABCG genes with 16 and 11 in *G. hirsutum* and the same in *G. barbadense*. Other than that, both Chr.5 of *G. arboreum* and Chr.9 of *G. raimondii* have the most ABCG genes, 12 and 11, separately.

**Figure 3:**
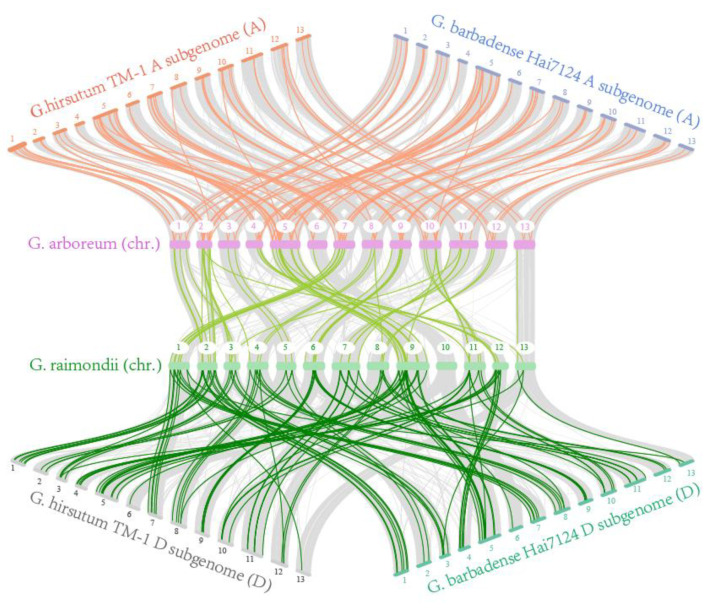
Collinear analysis of *ABCG*s among *G. hirsutum*, *G. barbadense*, *G. arboreum* and *G. raimondii*. Gray lines in the background indicate the collinear blocks and the colored lines highlight the syntenic *ABCG* gene pairs.

To investigate the evolution of the *GhABCG* gene family, we explored the gene duplication events. In total, 94 ABCG gene pairs were detected and all belong to segmental duplication, indicating that segmental duplication is the primary driver of *GhABCG* gene family expansion (Supplementary Table S3). We further conducted a genetic collinear analysis and found that 112 and 116 orthologous genes from *G. hirsutum* were found in *G. arboreum* and *G. raimondii*, respectively (Figure 3, Supplementary Table S4). However, 14 genes have no ortholog either in *G. arboreum* or *G. raimondii*, indicating that these genes can probably be yielded after a polyploidization event in *G. hirsutum*. Meanwhile, we also carried out collinear analysis between *G. barbadense* and its two ancestral species, *G. arboreum* and *G. raimondii,* and identified 111 and 113 orthologous genes in the two diploid species, respectively (Figure 3, Supplementary Table S4). In addition, 23 genes have no ortholog in the ancestral species. By calculating the ratio of the non-synonymous (Ka) and synonymous (Ks) substitution for each homologous gene pair in *G. hirsutum*, the selection pressures of *GhABCGs* were also investigated (Supplementary Table S5). In addition, the Ka/Ks ratio of all duplicated gene pairs is less than 1, implying that *GhABCG* genes have undergone purifying selection during the evolution process.

### 2.4. Cis-Acting Element Analysis

With the purpose of elucidating the putative function of GhABCGs in plant growth and development, the *cis*-acting elements in promoter regions of all *GhABCG* genes were investigated. Consistent with the results, the *GhABCG* promoters mainly contain the elements responding to hormones, stress and light. Hormone-response elements included the AuxRE and the TGA element responding to auxin, the CGTCA-motif and the TGACG-motif to MeJA, GARE-motif, P-box and TATC-box to gibberellin and TCA-element, and the ABRE and ERE to salicylic acid, ABA and Ethylene. Among them, the ABRE and ERE elements were more abundant in *GhABCG* genes, including 106 and 87, respectively. The abiotic stress-responsive elements included MYB and MBS responding to drought, LTR and MYC to low-temperature, ARE and GC-motif to anaerobic and TC-rich repeats to defense and stress, etc. According to the element analysis, 108 genes were supposed to be involved in both drought and low temperature stress, 107 genes responded to anaerobic and 53 responded to defense and stress. These findings indicated that *GhABCG* genes may exert vital functions in controlling plant growth and responding to abiotic stress.

### 2.5. Tissue Expression Pattern of GhABCG Genes

**Figure 4:**
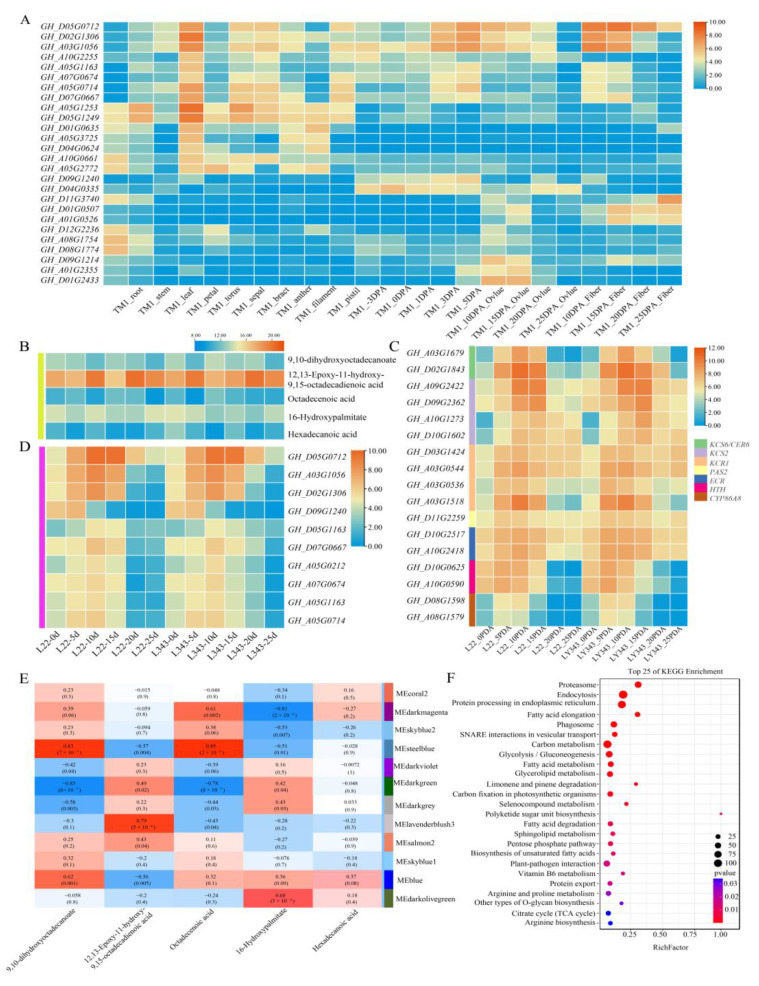
Expression and WGCNA analysis of *GhABCG* genes in developing fiber. (**A**) The expression pattern of 26 *GhABCG* genes in different tissues of upland cotton TM-1. (**B**) Quantitative analysis of five metabolites related to cutin biosynthesis in fibers of LMY22 and LY343 at different developmental stages. (**C**) A heat map showing the expression pattern of genes related to fatty acid elongation, cutin and wax biosynthesis in fibers of LMY22 and LY343 at different developmental stages. Seven genes are represented by vertical boxes with different colors, next to which the corresponding gene IDs are listed. (**D**) The expression pattern of 10 *GhABCG* genes in different developmental stages of fibers. (**E**) Transcriptome and metabolome combined analysis using the WGCNA R package. The numbers represent the correlation coefficient and *p*-value between the metabolites (rows) and the gene modules (columns). (**F**) KEGG pathway analysis of genes in the Medarkmagenta module. The size of the dots indicates gene numbers and the color of the dots represents the *p* value of pathway enrichment.

With the aim of further understanding the biological functions of *GhABCG* genes, the tissue expression pattern of these genes were explored based on online transcriptome data (root, stem, leaves, flower, ovule and fibers). As shown in Supplementary Figure S3A, *GhABCG*s exhibited different expression abundances among various organs and tissues. Twenty-six *GhABCG*s were highly expressed (TPM > 30) in at least one of the organs or tissues (Figure 4A, Supplementary Figure S3A). Eight of the 26 *GhABCG* genes were highly denoted in multiple tissues, for example, *GH_A03G1056*, *GH_D02G1306* and *GH_D05G0712* showed a high expression in petal, ovule and fiber. Seven *GhABCG* genes were highly denoted in root, stem and floral organs, including *GH_A05G1253*, *GH_D05G1249* and *GH_D01G0635*, etc. Interestingly, all the genes mentioned above have a relatively high expression in petals, such as *GH_A05G1253*, with the highest expression level (TPM ≥ 380). These *GhABCG*s high expression in multiple tissues implied their extensive functions during cotton growth. Typically, most of the *GhABCG* genes exhibited lower transcriptional abundance in the root, stem and especially in leaves compared to reproductive organs such as flower, ovule and fiber. Furthermore, some *GhABCG* genes were only highly expressed in a specific tissue or organ, for instance, *GH_A08G1754* and *GH_D08G1774* highly expressed in root, *GH_A05G1253* and *GH_D05G1249* highly expressed in stems, *GH_D09G1214*, *GH_A01G2355* and *GH_D01G2433* highly expressed in ovule, and *GH_D11G3740*, *GH_D01G0507* and *GH_A01G0526* highly expressed at late stage of fiber development. Different tissue expression patterns suggest that the substrate spectrum of *GhABCG*s may be more diverse, while tissue specific expression indicated the substrates of some *GhABCG*s may be specific.

### 2.6. Response of GhABCG Genes to Different Abiotic Stress

We explored transcriptome data of TM-1 under heat, cold, salt and PEG stresses to investigate the role of GhABCGs under a variety of abiotic stresses. The expression of almost half of *GhABCG* genes were triggered notably by one or more stresses (Supplementary Figure S3B). Obviously, *GhABCG* genes displayed stronger response to salts and PEG stresses than that to hot and cold stress, which may be due to different response mechanisms of the plant to osmotic stress (salts and PEG) and temperature stress (hot and cold). *GH_D05G1249*, *GH_A05G1253* and *GH_D02G2158* were significantly induced by four stress treatments. Interestingly, most *GhABCG* genes concurrently respond to salt and PEG stress, for instance, *GH_D01G1747*, *GH_A03G1056*, *GH_D05G0712* and *GH_A05G0714* were significantly induced under PEG and salt treatments, suggesting the important function of these GhABCGs in response to osmotic stress. However, *GH_D10G0709* and *GH_A10G0661* are significantly induced under hot treatments. The above results imply that GhABCGs may exert a vital function in the improvement of plant stress tolerance, conforming to the *cis*-acting element analysis of *GhABCG*.

### 2.7. Transcriptional Profile of GhABCGs in Developing Fiber

Cotton fibers are developed from the single seed epidermal cell and they are the primary raw material of textile industry. To study the potential roles of GhABCG transporters in fiber development, we analyzed the transcriptional profiling of 124 *GhABCG* genes using RNA-seq data from developing fiber of LMY22 (a high lint percentage cultivar) and LY343 (a high-quality variety). A total of 70 of the 124 *GhABCG* genes (56.5%) were expressed at one or more fiber development stages, with a wide expression level ranging from 1 to 776 tpm (tpm max) (Supplementary Figure S3C). Ten *GhABCG*s (8.1%) have a higher expression (TPM > 30) in developing fibers and were mainly highly expressed during the fiber elongation period (5–15 day post-anthesis, DPA) (Figure 4D). However, one gene (*GH_D09G1240*) was highly expressed in a mixture of fiber and ovule at 0 and 5 DPA (when fiber begins to initiate, fiber and ovule cannot be separated), suggesting that *GH_D09G1240* may function in young ovules. Three (*GH_D05G0712*, *GH_A03G1056* and *GH_D02G1306*) *GhABCG*s had a higher expression level and reached a maximum at 10 DPA, suggesting the three genes may actively translocate compounds to support the elongation of fibers. Although expression levels of these *GhABCG* genes showed slight difference between LMY22 and LY343, the expression trends in both varieties were similar.

### 2.8. WGCNA Analysis of Transcriptome and Metabolome in Developing Fiber

Our transcriptome data showed that *GH_D05G0712* (*GhABCG15*), *GH_A03G1056* (*GhABCG11_A03*) and *GH_D02G1306* (*GhABCG11_D02*) were highly expressed in developing fiber from 0 to 20 DPA. It has been reported that only ABCG subfamily members were engaged in cuticle formation through exporting cutin precursors such as lipids and hydrophobic compounds [14]. To better explore the functional roles of highly expressed *GhABCG*s in fiber development, we analyzed the aliphatic metabolites involving in cutin biosynthesis in developing fibers. Five metabolites (16-Hydroxypalmitate, 9,10-dihydroxyoctadecanoate, 12,13-Epoxy-11-hydroxy-9,15-octadecadienoic acid, Octadecenoic acid, Hexa-decanoic acid) enriched in cutin and the wax biosynthesis pathway were picked for further analysis (Figure 4B).

It was found that the five metabolites were differentially accumulated in the fiber at initiation (0–5 DPA), elongation (5–15 DPA) and second wall formation (15–20 DPA). We performed a combined transcriptome and metabolome analysis using the WGCNA R package. As shown in Figure 4E, the Medarkmagenta and MEsteelblue modules had a higher correlation (|r| > 0.8) with the five lipid metabolites. Among these, the Medarkmagenta module was significantly negatively associated with 16-Hydroxypalmitate and the MEsteelblue module was positively related to dihydroxy-octadecenoate and octadecenoic acid. Nine highly expressed GhABCG genes (including *GhABCG15*, *GhABCG11_A03* and *GhABCG11_D02*) are clustered in the Medarkmagenta module (Supplementary Table S6), suggesting *GhABCG* genes’ transport activity may negatively correlated with the amount of major cutin precursor. As the expression peak of the cutin-precursor-synthesis related gene were present earlier compared to that of three *ABCG* genes, the negative correlation meant that a large number of cutin precursors were exported to the outermost layer of the fiber cell wall for cutin polymerization, leading to the reduction of cutin precursors. KEGG analysis showed that genes in the Medarkmagenta module were enriched in lipid metabolism related pathways such as fatty acid elongation (24 genes), fatty acid metabolism (33 genes), glycerol-lipid metabolism (35 genes), sphingolipid metabolism (15 genes), biosynthesis of unsaturated fatty acids (13 genes) and fatty acid degradation (22 genes) (Figure 4F). The results implied that GhABCG transporters may exert vital functions in the cutin formation of cotton fiber through exporting lipid metabolite.

Further, we investigated other differentially expressed genes in fatty acid elongation and the cutin, suberin and wax biosynthesis pathway between fiber developmental stage of LMY22 and LY343, including *KCS2*, *KCS6*, *KCR1*, *PAS2* and *ECR*, involved in VLCFA biosynthesis, as well as *HTH* and *CYP86A8*, involved in cutin precursor biosynthesis in *Arabidopsis* (Yeats and Rose, 2013). Based on the obtained results, these investigated genes have similar expression pattern, with *GhABCG15*, *GhABCG11_A03* and *GhABCG11_D02* up-regulating in 5-, 10- and 15-DPA fibers and then declining within the subsequent fiber developmental stages (Figure 4C), implying that these *GhABCG* genes actively contribute to export aliphatic composition for fiber development such as fiber cutin formation. However, limited data could not completely exclude these GhABCGs participating in other biological processes, such as fiber elongation and secondary wall thickening.

### 2.9. Silencing of GhABCG15 Altered Leaf Morphology and Cuticular Structure

**Figure 5:**
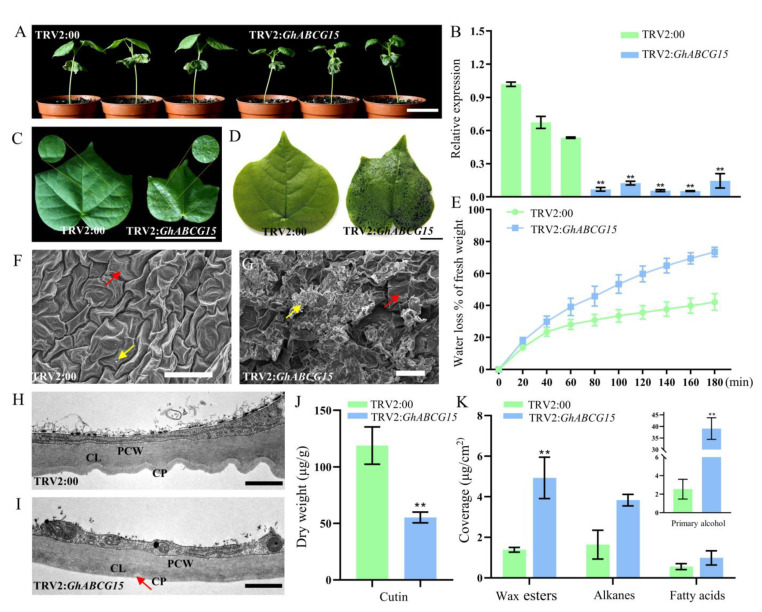
The phenotype and cuticular composition of *GhABCG15*-silenced plant leaves. (**A**) The phenotype of the cotton seedlings after *GhABCG15* was silenced by VIGS for 10 days. TRV2:00 and TRV2:*GhABCG15* represent control plants injected with empty vector and *GhABCG15*-silenced plants injected with *GhABCG15* fragments (similarly hereinafter). Scale bar represents 5 cm. (**B**) The *GhABCG15* expression levels in the leaves of control plants and *GhABCG15*-silenced plants were detected using qRT-PCR (Mean ± SD, n = 3; Student’s *t*-test, ** *p* < 0.01). (**C**) Leaf morphology observation of control and *GhABCG15*-silenced cotton seedlings, scale bar represents 4 cm. (**D**) Toluamide-blue staining of the whole leaves of control and *GhABCG15*-silenced cotton. Scale bar represents 1 cm. (**E**) Water loss rate detection of the detached leaves of control and *GhABCG15*-silenced cotton (Mean ± SD, n = 3). (**F**,**G**) show epidermal surface of control and *GhABCG15*-silenced leaves respectively obtained by scanning electron microscopy. Red arrow shows cuticular ridges and yellow arrow indicates epicuticular wax. Scale bars represent 50 μm. The cuticle ultrastructure of leaves cross-section of control (**H**) and *GhABCG15*-silenced cotton (**I**) were observed via transmission electron microscopy (TEM). Red arrow in H denotes the rupture point in cuticle structure of *GhABCG15*-silenced cotton. Scale bars in H and I represent 10 μm. (**J**) Cutin component quantification in control and *GhABCG15*-silenced cotton leaves (Mean ± SD, n = 3; Student’s *t*-test, ** *p* < 0.01). (**K**) Wax monomer components analysis in control and *GhABCG15*-silenced cotton leaves (Mean ± SD, n = 3; Student’s *t*-test, ** *p* < 0.01). CP: Cuticle Proper; CL: Cuticular Layer; PCW: Polysaccharide Cell Wall.

To explore the function of *GhABCG15* in cotton, a VIGS system was employed to knock-down the expression of *GhABCG15* using the TRV2 vector (TRV2: *GhABCG15*). After infection for ten days, the plants with TRV2:*GhCLA1* presented the albino phenotype, suggesting that the VIGS system worked well. qRT-PCR analysis revealed that the expression of *GhABCG15* was notably reduced in *GhABCG15*-silenced plants relative to the controls (Figure 5B). Phenotype identification showed that *GhABCG15*-silenced plants became dramatically smaller than the control, with crinkled leaves (Figure 5A). Three weeks later, the leaves of *GhABCG15*-silenced plants displayed some glossy depressions and punctate patterns of distribution (Figure 5C). Toluamide-blue staining of detached leaves indicated that indentations in *GhABCG15*-silenced leaves are more strongly stained (Figure 5D) and water loss rate tests showed that the percentage of water lost in the *GhABCG15*-silenced leaves was higher than the control (Figure 5E), suggesting that the permeability of the cuticle had increased.

To determine if silencing of *GhABCG15* leads to alterations in epicuticular wax structure, this study explored the adaxial surface of leaves by adopting scanning electron microscopy (SEM). The results presented a significant difference in the distribution and density of wax crystals between the silenced plants and control and the ruptures appeared in the *GhABCG15-*silenced leaf epidermis (Figure 5F,G). Interestingly, the cuticular surface of controls had a narrow ridge, but the *GhABCG15*-silenced leaves showed fewer bulges and had partly cuticular ridges which became wider and flatter. Moreover, a large accumulation of epicuticular wax was found on the leaf surface of silenced plants, though plate-like crystals were not uniformly distributed over the epidermis. However, a small amount of wax evenly covered the leaf surface of control plants. To further determine whether there was an alteration in cuticle structure, we observed the ultrastructure of leaf crosscut with the use of transmission electron microscopy (TEM). The findings demonstrated that the leaf cuticle of the control had a continuous and regular layered structure, but in *GhABCG15*-silenced plants the outermost cell wall boundary is protected by a minimal amount of the electron-dense overlay (Figure 5H,I). In addition, empty spaces are occasionally observed, implying that the cuticle structure of *GhABCG15*-silenced plants was discontinuous.

We further examined the cuticular wax and cutin component of leaves in the *GhABCG15*-silenced plants as well as the control by adopting Gas Chromatography-Mass Spectrometry (GC-MS). In addition, the wax load (aliphatic series) in *GhABCG15*-silenced plants was significantly increased by seven times compared to the controls (Figure 5K). Primary alcohol, the most dominant component of wax, increased by fourteen times, resulting in an increase in wax load. Next, we analyzed the aliphatic cutin monomers to examine whether the alteration in cuticle ultrastructure was accompanied by changes in the deposition of cutin. According to the obtained findings, the incorporation of aliphatic cutin monomers into cutin was more strongly lowered in *GhABCG15*-silenced plants than the controls (Figure 5J). Collectively, the above results suggested that *GhABCG15* may involve in cuticle formation by exporting cutin precursors.

## 3. Discussion

The ATP Binding Cassettte (ABC) protein family is a large and widespread gene family [31]. Studies have shown that ABC transporters have vital functions in the protection of the plant from endogenous and exogenous toxic compounds and they are also fundamental for normal development of plants in adapting to a sessile lifestyle. A genome-wide identification of the ABCG subfamily is clearly warranted by the fact that it is the largest subfamily in the ABC family and the substrates transported by ABCGs tend to be secondary compounds of terpenoid origin and highly lipophilic compounds [6]. The ABCG gene family has been featured in several plant species containing *Arabidopsis thaliana*, *Oryza sativa* and *Capsicum spp*. [32,33,34]. However, the research on *Gossypium* ABCG gene family has been comparatively rare until now. In the current work, we identified 395 ABCG family members from *G. arboreum* (69 genes), *G. raimondii* (71 genes), *G. hirsutum* (124 genes) and *G. barbadense* (131 genes). Phylogenetic analysis suggested that the *Gossypium* ABCG proteins were classified into four groups belonging to two types: type I (Ia, Ib, Ic) belong to White Brown Complex (WBC) and type II belong to Pleiotropic Drug Resistance (PDR), respectively. In *Arabidopsis*, multiple sequence analysis showed that the ABCG subfamily includes 29 half-size transporters (WBC) and 15 full-size transporters (PDR) [35,36]. Multiple species sequence analysis of ABC genes confirmed that half- and full-size transporters have evolved from the same ancestor and the PDR transporters may be derived from duplication of a half-size transporter gene [36]. The insertion/deletion events can modify the gene structure and this alteration could cause the gene to evolve a new function [37]. Gene structure and motif composition analyses suggested that most *GhABCG*s have similar exon-intron structures in the same clade and motif distributions and irregular distribution among various clades, suggesting the protein structures were associated with functional differentiation. *G. hirsutum*, as an allotetraploid, was putatively originated from a natural interspecific hybridization between the A-genome diploid *G. arboreum* and D-genome diploid *G. raimondii* [38]. Gene duplication is an important means to generate new genes and functions. The gene duplication can be expanded in four primary modes: whole genome duplication, segment duplication, tandem duplication and gene transposition duplication [39,40]. In our study, all duplicated gene pairs belong to segmental duplication which is the leading reasons for *ABCG* gene family expansion of two allotetraploids. The number of copies of most genes between diploid and tetraploid is in one-to-two correspondence. However, some genes in both allotetraploids could not be collinear with diploids, such as *GH_D11G2292*, *GH_D11G1107*, *GB_A01G1740* and *GB_A02G2084,* etc., suggesting that these genes may be newly created copies after gene duplication, or errors in genome assembly. We found that *GhABCG* genes are subject to purification selective pressure. In addition, purification selection masking deleterious mutations may increase the chance of neofunctionalization during the evolution of *GhABCG* genes [41].

Much research has indicated that ABCG proteins are engaged in numerous plant developmental processes including pollen development [42,43], lignin synthesis [44], suberin formation [45,46], cuticle formation [13,47], pathogen response and plant defense [48,49], and hormone transport [50,51]. Promoter cis-acting element analysis demonstrated that promoters of *GhABCGs* contained several cis-elements responding to plant hormones, such as ABRE, ERE and CGTCA-motif, and to abiotic stress, such as MYB and MYC, indicating a potential role of *GhABCG* genes in cotton growth and development as well as abiotic stress response. For example, *GH_D05G1249*, *GH_A05G1253* and *GH_D02G2158* are significantly induced by four stress treatments, while *GhABCG15* (the homologous genes of *AtABCG15*), *GhABCG11_A03* and *GhABCG11_D02* (the homologous genes of *AtABCG11*) were predominantly expressed in petal, ovule and fiber. In *Arabidopsis*, CER5/ABCG12 and ABCG11 are required for cuticle development with the transport of lipids and hydrophobic compounds [13,24]. Although CER5/ABCG12 shares 85% identity with AtWBC15 in the amino acid level [23], there was no direct evidence provided for wax transport of AtWBC15. Additionally, some genes co-expressed with these lipid-transfer genes and their homologs have been documented to be involved in cuticular wax and cutin biosynthesis in *Arabidopsis*, including *GhKCS2*, *GhKCS6*, *GhKCR1*, *GhPAS2*, *GhECR*, *GhHTH* and *GhCYP86A8* [14]. Therefore, we analyzed metabolic profiling of cotton developing fiber and found that five metabolites (9,10-dihydroxyoctadecanoate; Octadecenoic acid; 16-Hydroxypalmitate; Hexa-decanoic acid; 12,13-Epoxy-11-hydroxy-9,15-octadecadienoic acid) were enriched in the cutin and wax biosynthesis pathways. WGCNA analysis of transcriptome and metabolome from fiber showed that *GhABCG15*, *GhABCG11_A03* and *GhABCG11_D02* were clustered in the Medarkmagenta module and had a higher negative correlation with 16-Hydroxypalmitate, indicating that the expression pattern of *GhABCG15* in cotton fiber may be significantly correlated with 16-Hydroxypalmitate content. Furthermore, 16-Hydroxypalmitate was a conjugate base of a 16-hydroxyhexadecanoic acid that was one of the leading cutin monomers of *Arabidopsis* stem and leaf epidermis [14]. The significant correlation implied that the three GhABCG transporters may be engaged in transport of cutin precursor, which contributes to cutin formation of fiber.

With the purpose of understanding the biological function of genes from the Medarkmagenta module, KEGG pathway enrichment analysis was performed. Six KEGG pathway related to lipids metabolism were enriched in the top 25 terms, containing fatty acid elongation, fatty acid metabolism, glycerol-lipid metabolism, sphingolipid metabolism, biosynthesis of unsaturated fatty acids and fatty acid degradation. On the basis of all the above results, we speculate that GhABCGs may be engaged in the transport of aliphatic precursor during cotton fiber development.

The plant cuticle plays a role of barrier against water loss through transpiration, defense against pests and pathogens, screening excessive UV light, self-cleaning the surface and establishing organ boundaries. The ABCG subfamily is essential for the transport of cuticular lipids in many plant species, containing monocots and dicots [13,52,53,54]. CER5/AtABCG12 was first documented to be involved in transmembrane transport of wax in the cuticle of the stem epidermis. With the process of mass spectrometry technology, a large number of cutin monomers and wax components have been detected. Although the transport of wax and cutin precursors across the plasma membrane depends on ATP-binding cassette (ABC) transporters, most of the transport processes are not well understood. Initially, a glossy stem was used as a criterion to screen wax-deficient mutants [55]. It has been reported that the proportion of alkanes, rather than their total amount, are critical determinants of cuticle permeability in *Capsicum annum* (high postharvest water loss rates) and *Capsicum chinense* (low postharvest water loss rates) [56]. Cutin deficiencies, which cause tissue defects, are harmful to cuticle water permeability [28]. However, there is no association with cuticle water permeability and cuticle thickness [57]. In this study, the *GhABCG15*-silenced leaves display a crinkled phenotype with many glossy indentations on the leaf epidermis. Meanwhile, *GhABCG15*-silenced plants had an increased cuticular water permeability, when measured with the use of toluidine blue staining and water loss. The changes in leaf morphology and cuticle permeability indicated an alteration in the proportion of certain waxy components or cuticle monomers. The PEC1/ABCG32 transporter is vital for transport of cutin monomers as well as the formation of regular cuticular ridges in petals [28,54]. The phenotypes of *osabcg31* and *OsABCG31*-RNAi plants elevated cuticle permeability, lowered the amount of cutin and altered the cuticle ultrastructure [58]. In the current work, scanning electron microscopy of leaf epidermis showed an increase in epicuticular wax crystals and a loss of cuticular ridges, and transmission electron microscopy showed a thinner and discontinuous cuticle layer. *Arabidopsis thaliana* has been identified as a vital model for deciphering the pathway of cutin biosynthesis, with the main component being a dicarboxylic acid in the stem and leaves, but more typically 10,16-dihydroxyhexadecanoic acid in the floral organs [59,60]. The *AtABCG11* knockout mutant showed a 75–90% reduction in alkanes and a 65% reduction in total cuticle monomers, resulting in severe dwarfing, a permeable cuticle and potential organ fusions [12,13]. Consistent with these results, in our studies *GhABCG15*-silenced leaves exhibited significant changes in wax and cutin composition compared to the WT. Cutin was dramatically reduced in *GhABCG15*-silenced plants. However, wax composition was significantly increased in the *GhABCG15*-silenced plants, especially primary alcohols, indicating a compensatory impact for the loss of cutin components. In *Arabidopsis*, cuticle-associated half transporters were assembled into homodimer or heterodimers to transport different compound classes [61]. Similarly, *GhABCG15* may also form either a homodimer or a heterodimer to transport different cutin monomers. However, it is necessary to conduct future research to verify the role of *GhABCG15* in cotton cutin formation.

## 4. Materials and Methods

### 4.1. Sequence Retrieval and Identification of GhABCGs

Genome sequence and information of four *Gossypium* spp. were acquired from the CottonFGD (https://cottonfgd.net/) (accessed on 4 January 2022) [62], including *G. hirsutum* (Ver. ZJU 2.1), *G. barbadense* (Ver. ZJU 2.1), *G. arboretum* (Ver. CRI 1.0) and *G. raimondii* (Ver. JGI 2.0). We downloaded a total of 43 ABCG genes of *A.thaliana* from the TAIR (Ver.TAIR 10) (https://www.arabidopsis.org/) (accessed on 4 January 2022) database. In addition, based on the hmmbuild command of HMMER v3.3.2 software, the Hidden Markov Model (HMM) was constructed from 43 *Arabidopsis* ABCG proteins and was employed to search for the corresponding protein sequences; meanwhile, we applied full-length amino acid sequences in the *Arabidopsis* ABCG proteins as queries for searching corresponding protein sequences by BLASTP. We selected proteins with a homology of least 30% similarity and an e-value less than e^−15^. To further prove the accuracy of the candidate *ABCG* gene, we examined present of TMD-NBD or TMD-NBD-TMD-NBD in ABCG proteins with SMART (http://smart.embl-heidelberg.de/) (accessed on 12 January 2022) (Letunic et al., 2021) and Pfam (http://pfam.xfam.org) (accessed on 12 January 2022) (Mistry et al., 2021). Then genes without the above-mentioned structures were removed by manually checking. Based on the web-based ExPasy approaches (http://web.expasy.org/protparam/) (accessed on 17 January 2022), we estimated length, isoelectric point (pI) and molecular weight (MW) for GhABCG proteins. Meanwhile, for the purpose of predicting subcellular locations, we adopted CELLO v2.5 server (http://cello.life.nctu.edu.tw/) (accessed on 17 January 2022).

### 4.2. Phylogenetic Analysis

Using Cluster X software with default parameters, multiple-sequence alignments were conducted using the sequences of GhABCG protein in *Arabidopsis* and four *Gossypium* spp. Then, iqtree software was adopted for constructing phylogenetic trees in line with the findings of multiple sequence alignment, using Maximum likelihood (ML) with the JTT + R7 model along with 1000 bootstraps. Eventually, online ITOL (http://itol.embl.de/) (accessed on 18 January 2022) was applied in establishing a phylogenetic tree.

### 4.3. Gene Structure Analysis and Conserved Motif Identification

For inquiring into their exon-intron organizations, genome sequences of *ABCG* along with the corresponding CDS sequences collected in cotton (*G. hirsutum*) genome were imported into Gene Structure Display Server (http://gsds.cbi.pku.edu.cn/) (accessed on 20 January 2022) [63]. The web-based tool Motif Elicitation (MEME) (http://meme.sdsc.edu/meme/intro.html) (accessed on 20 January 2022) was adopted for detecting conserved motifs within ABCG proteins [64]. Furthermore, Tbtools was applied in annotating those detected protein motifs [65].

### 4.4. Chromosomal Location, Gene Evolutionary and Cis-Acting Element Analyses

The chromosomal location information of *ABCG* genes was acquired from cotton genome database (https://cottonfgd.net/) (accessed on 24 January 2022) and mapped using Tbtools. On the basis of the principle described in the former report, gene duplication events of *ABCG*s were identified [66]. Furthermore, gene duplicates and chromosomal locations were visualized using the Circos-0.69 Software [67]. Orthologous gene pairs among genomes of upland cotton or sea-island cotton with the ancestral A/D diploid cotton species were detected by Blast version 2.2.9 [68]. Then, McscanX was used to perform whole genome collinear analysis [69]. Lastly, with the use of the KaKs calculator v 2.0, synonymous and non-synonymous rates of evolution were calculated [70].

GhABCG gene promoter sequences (2000-bp from upstream initiation codon “ATG”) [71] were imported into Plant Care database (https://bioinformatics.psb.ugent.be/webtools/plantcare/html/) (accessed on 26 January 2022) for promoter element prediction.

### 4.5. RNA-Seq and Metabolome Analysis

This work obtained corresponding transcriptomic results on *G. hirsutum* acc. TM-1 at NCBI (BioProject PRJNA490626) and fiber transcriptome data of LMY22 and LY343 from our lab (PRJNA546484). Raw reads quality assessment was carried out with the use of the fastp software [72]. We mapped RNAseq reads against reference genome of cotton (v2.1) based on hisat2. Thereafter, gene expression profiles (Supplementary) were quantified by feature Counts [73].

Tbtools was used to draw the heatmap of *GhABCG* gene expression profiles [65]. This work was carried out WGCNA with WGCNA R package [74] and the soft power threshold was set to 8. Untargeted metabolomics was performed with ultra-performance liquid chromatography (UPLC) (Shimazu, Kyoto, Japan) coupled to the high-resolution tandem mass spectrometer TripleTOF 6600 (SCIEX, Framingham, MA, USA) at Hangzhou Lianchuan Biotechnology Co., Ltd. (Hangzhou, China).

### 4.6. Plant Materials and Growth Conditions

Hydrogen peroxide (Sinopharm, Shanghai, China) (10% (*v/v*)) was added to soften cotton (*G. hirsutum* cv. LMY 37) seeds obtained from our lab for 2h and rinsed in sterile water. Then, the treated seeds were covered with damp gauze for 12–14 h, followed by pot planting with consistent buds, then growing based on controlled situations (25 °C, 16-h/8-h light/dark). In addition, we adopted cotton seedlings that had completely expanded cotyledons for virus-induced gene silencing (VIGS) infiltration after around 7-days growth.

### 4.7. Vector Construction and Genetic Transformation

The pTRV1 and pTRV2 vectors were used in the VIGS experiment which preserved in our laboratory. A 308-bp specific fragment was obtained from cDNA by amplification based on homologous arm primer pairs including restriction sites Kpn1. Meanwhile, the pTRV2 vector was linearized with Kpn1 (TaKaRa, Dalian, China). The aforementioned products were purified by Tiangen^®^ PCR products purify kits (Tiangen, Beijing, China). An amplified sequence was obtained from cDNA by amplification based on primer pairs including restriction enzyme recognition sites. With ClonExpress^®^ II One Step Cloning Kit (Vazyme, Nanjing, China), that gene fragment was cloned into pTRV2. After PCR and Sanger sequencing confirmation, the TRV2:ABCG15 construct was transformed into Agrobacterium tumefaciens strain GV3101 with the application of the freeze-thaw method. According to previous reports, the VIGS assay was performed [66].

### 4.8. qRT-PCR Analysis

Using FastPure^®^ Universal Plant Total RNA Isolation Kit, we extracted total RNA of the collected cotton leaves (Vazyme, Nanjing, China). Subsequently, with the application of HiScript^®^ II 1st Strand cDNA Synthesis Kit (Vazyme, Nanjing, China), first-strand cDNA was synthesized in line with specific instructions. The present work adopted synthesized cDNA to be template in qRT-PCR conducted by Light Cycler 480 (Roche, Basel, Switzerland) with ChamQ Universal SYBR qPCR Master Mix (Vazyme, Nanjing, China). Supplementary Table S8 lists the primers adopted in the current work.

### 4.9. Determination of Wax and Cutin Composition

Cuticular wax was obtained by extracting the second real leaf using the chloroform extraction method, as depicted elsewhere [75]. The sample was soaked within the chloroform, followed by 60-s slow shaking with the purpose of removing cuticular waxes. The extracts were added to 100 μg n-octadecane (C18 alkane) (Sigma Aldrich, Shanghai, China) as the endogenous reference. Crude extract of all samples was dissolved and dried, repeated twice.

Pyridine (100 μL, Fluka, Seelze, Germany) was added to dissolve residues, followed by derivatization by sylation using bis-N,N-(trimethyl-silyl) tri-fluoracetamide (100 μL, BSTFA, Sigma Aldrich, Shanghai, China) for 50 min at 70 °C. The derivative reagent was dissolved in chloroform for GC-MS analysis. Then, the SH-I-5MS column (0.25 mm × 0.25 μm × 30 m, Shimadzu, Kyoto, Japan) connected with the mass spectrometer (GCMS-QP2010 SE, Shimadzu, Kyoto, Japan) was adopted for exploring cuticular wax composition. Initial oven temperature was 50 °C for 2 min, followed by ramping at 20 °C min^−1^ till 200 °C, 2-min holding under 200 °C, ramping at 2 °C min^−1^ till 320 °C and 14-min holding under 320 °C. To perform the subsequent analysis, the data were normalized by total peak area.

The cutin extraction was performed with reference to Yang et al. [75]. Cotton leaves were collected and weighed in the same fashion as described above in wax extraction for GC–MS. All plant samples were immersed in isopropanol, followed by 10-min incubation in the 85 °C water bath. Next, we added to the sample tube again 20 mL of isopropanol, shaken overnight. Finally, two chloroform/methanol (2:1, *v/v*) extractions and one methanol extraction were performed in sequence for 24 h each. A solid residue was dried in a fume hood at the nitrogen atmosphere overnight and maintained for three days in a desiccator. Then, the ester bond of the keratin compound is broken and the fatty acid in the stratum corneum is methylated under the action of sodium methanol. The processed samples were analyzed qualitatively and quantitatively by GC-MS.

### 4.10. Cuticle Permeability Assays

The toluidine blue staining was carried out following the previous description [53], with 0.05% toluidine blue solution for 45 min and subsequently rinsed several times with deionized water. Pictures were taken with a digital camera.

For measuring water loss, the second real leaf were gathered and immersed in the dark for a 1-h period within deionized water. The excess liquid was wiped dry by paper towel. The leaves were again kept away from light. Then, leaf sample weights were measured at 0 min, 20 min, 40 min, 60 min, 80 min, 100 min, 120 min, 140 min, 160 min and 180 min. The water loss rate was determined by lost water wight/fresh weight. The above procedure was carried out on the basis of the method proposed by Yang et al. [75].

### 4.11. Electron Microscopy

The second real leaf of the *GhABCG15*-silencing cotton plants with cuticle defect and wild type controls were collected and dried at room temperature for one week. After complete drying, based on a field emission scanning electron microscope, the dried plant materials were evaluated (FEI, Quanta™ 450, Hillsboro, OR, USA).

In order to observe the cuticle membrane ultrastructure, the second real leaf was made into ultrathin sections following the previous method [53]. Micrographs were taken using a transmission electron microscope HT7800 (Hitachi, Tokyo, Japan).

### 4.12. Statistical Analysis

Each experiment was tested for three biological replicates in triplicate. Data were shown as the mean value ± SD (standard deviation).

## 5. Conclusions

To conclude, this work carried out a genome-wide identification and characterized ABCG transporters in four *Gossypium* spp. According to the results, the gene structures of *GhABCG*s are both conservative and diverse. WGCNA analysis showed nine highly expressed *GhABCG* genes were significantly associated with the amount of main cutin precursor. Silencing of *GhABCG15* via VIGS resulted in decreased leaf cutin content and thus reduced thickness of cuticle, suggesting that the *GhABCG15* involved in cuticle formation may by transporting cutin precursors in cotton leaves. Moreover, the findings will lay a basis for exerting the impact of *ABCG* genes on cotton growth and development.

## Data Availability

The raw transcriptome data from our lab was submitted to the NCBI-SRA database under the project id: PRJNA546484.

## Supplementary Materials

The following supporting information can be downloaded at: https://www.mdpi.com/article/10.3390/ijms24032379/s1.
